# Hexavalent chromium waste removal *via* bioelectrochemical systems – a life cycle assessment perspective†

**DOI:** 10.1039/d3ew00344b

**Published:** 2023-07-31

**Authors:** Rukayya Ibrahim Muazu, Jhuma Sadhukhan, S. Venkata Mohan, Siddharth Gadkari

**Affiliations:** a Centre for Environment & Sustainability (CES), School of Sustainability, Civil and Environmental Engineering, University of Surrey Guildford GU2 7XH UK r.ibrahim@surrey.ac.uk; b Bioengineering and Environmental Sciences Lab, CEEFF Department, CSIR-Indian Institute of Chemical Technology (CSIR-IICT) Hyderabad 500 007 India; c School of Chemistry and Chemical Engineering, University of Surrey Guildford GU2 7XH UK s.gadkari@surrey.ac.uk

## Abstract

Bioelectrochemical systems (BESs) such as microbial fuel cells (MFCs) present numerous benefits for the removal and recovery of heavy metals from industrial and municipal wastewater. This study evaluated the life cycle environmental impact of simultaneous hexavalent chromium (Cr(vi)) removal and bioelectricity generation in a dual chamber MFC. Results indicate a global warming potential (GWP) of −0.44 kg carbon dioxide (CO_2_)-eq. per kg of chromium recovered, representing a total saving of up to 97% in comparison with existing technologies for the treatment of Cr(vi) laden wastewater. The observed savings in GWP (kg CO_2_-eq.) reduced to 61.8% with the removal of the allocated credits from the MFC system's life cycle. Of all the various sub-systems considered within the chromium waste treatment plant, the MFC unit and the chromium metal recovery unit had the largest impact in terms of GWP (kg CO_2_-eq.), non-renewable energy use (NREU) (MJ primary), and mineral extraction (MJ surplus). A statistical analysis of the results showed that an increase in chemical oxygen demand (COD) was associated with a reduction in GWP (kg CO_2_-eq.), NREU (MJ primary), and terrestrial ecotoxicity (kg triethylene glycol equivalents into soil (TEG soil)-eq.). The life cycle assessment (LCA) output showed a high sensitivity to changes in the materials and construction processes of MFC reactors, indicating the need for further research into sustainable materials for MFC reactor construction. The observed interaction effects of process variables also suggest the need for combined optimization of these variables. Analysis with other types of metals is also important to further demonstrate the practical viability of metal removal through MFCs.

## Abstract 2

First LCA study for assessing the environmental performance of BESs for metal recovery application.

## Graphical abstract

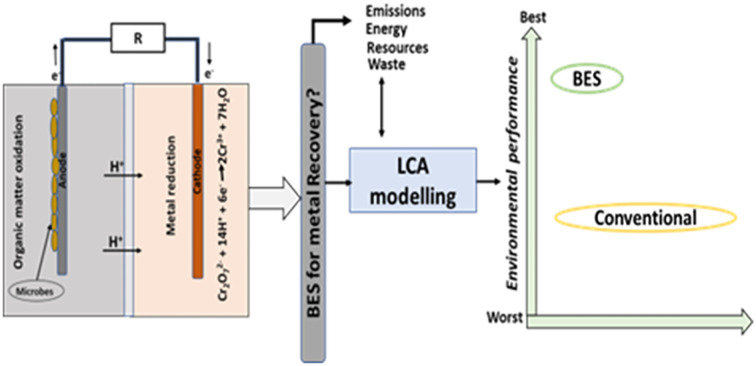

**Water impact**

Bioelectrochemical systems (BESs) have the potential to reduce environmental pollution and minimize the carbon footprint of metal recovery processes from wastewater. Conducting a comprehensive LCA helps identify and mitigate any negative impacts associated with BES application on a larger scale. This work is essential for ensuring the long-term viability of BESs as a sustainable and eco-friendly approach for metal recovery.

## 1. Introduction

Heavy metals have detrimental effects on human health, and the generation of heavy metal-laden waste streams has increased due to various industrial activities such as mining, metallurgical operations, burning fossil fuels, manufacturing, and batteries.^1^ Water, air and food contamination by toxic metals such as chromium, cadmium, nickel, mercury and lead is an environmental and health concern that affects millions around the world.^2,3^

Chromium metal is commonly used in various industrial processes including metal electroplating, leather tanning, metallurgy, dye manufacturing and corrosion control in cooling towers.^1,4^ Unlike some organic pollutants, chromium metal and some heavy metals are non-biodegradable and can remain in the ecosystem for a long time.^5^ The two most stable forms of chromium found in the environment are hexavalent Cr(vi) and trivalent Cr(iii). Cr(vi) is characterised by high solubility, mobility and toxicity, and as a mutagen, carcinogen and teratogen, while Cr(iii) is non-toxic, highly distributed in insoluble mineral, and is even regarded as an essential microelement for organisms at low concentrations.^5,6^ Meanwhile, toxic forms of hexavalent chromium (Cr(vi)) such as chromate and dichromate are present in wastewater from tanneries, electroplating and other chemical plants with concentrations as high as 1000–1524 mg L^−1^ of Cr(vi).^7^ Cr(vi) can also find its way to the food chain through chromium contaminated drinking water posing a serious risk to human health and the ecosystem. Water contamination by chromium (Cr) has attracted great attention in the present context of global ecology because of the extensive use of this metal in textile industries, electroplating, leather processing, metal finishing, and chromium preparation.^3,8,9^

Considering the adverse effects, regulation of hexavalent chromium has been incorporated in various Directives of the European Union, such as the RoHS Directive (Restriction of the Use of Certain Hazardous Substance in Electrical and Electronic Equipment)^10^ or the ELV (End-of-Life Vehicles).^11^ The U.S. Environmental Protection Agency has identified Cr(vi) as one of the seventeen chemicals causing the largest threat to human health, thus, a maximum concentration limit for discharge has been set as 50 μg L^−1^.^9,12^

Over the years, various methods such as adsorption, membrane technology, chemical, electric, and photocatalytic-based treatments have been used to treat metal laden wastewater from different sources.^8,13,14^ While there have been noteworthy achievements, it should be noted that each approach to heavy metal removal has its own set of challenges, encompassing cost, efficacy, and the production of additional waste. These factors have collectively impacted the comprehensive success of these methods. Recent interest has been directed towards bioelectrochemical systems (BESs) including microbial fuel cells (MFC) and microbial electrosynthesis (MES) for hexavalent chromium removal from wastewater.^1,4,15,16^

BESs such as MFCs use microorganisms as a biocatalyst and convert chemical energy from organics in wastewater to electrical energy.^9,17,18^ It is a technology for simultaneous removal of chemical oxygen demand (COD) in wastewater in the anode chamber and synthesis of products in the cathode chamber by adopting the principle of oxidation and reduction (redox) reactions and providing an electric field between an anode (positive electrode) and a cathode (negative electrode).^19,20^ Meanwhile in MES, electrogenic bacteria harvest electrons utilizing organic or impurities present in wastewater as a substrate in the anode chamber, while compounds such as carbon dioxide/(bi)carbonate ions can be reduced in the cathode chamber by consuming harvested electrons from the anode chamber to synthesize volatile fatty acids, producing valuable chemicals such as formic acid, acetic acid, propionic acid, butyric acid, valeric acid and caproic acid in MES.^21,22^ The application of MFC technology holds great promise for the removal of heavy metals from wastewater, owing to its distinct ability to facilitate the production of electricity while simultaneously eliminating toxic pollutants.^23^ Metal ions such as hexavalent chromium (Cr^6+^) can be reduced to trivalent (Cr^3+^) in the cathode chamber by consuming harvested electrons from the anode chamber, and further precipitating the non-toxic chromium Cr^3+^, thus recovering the high purity low toxicity metal.^4^ Several studies have explored various approaches for chromium metal removal in MFCs, and factors influencing the efficiency of Cr(vi) reduction such as the concentration and composition of the wastewater, the organic substrates, the type of electrode, the pH of the electrolytes, the stability of the membrane/separator and the microorganism, have also been investigated.^4,6,24^ Carbon electrodes have been more widely utilised in MFCs for Cr metal removal; among these, carbon cloth has high electronic conductivity and flexibility compared with felt and brushes.^5^ Meanwhile the use of an α-Fe_2_O_3_/polyaniline nanocomposite as the cathode improved power density generation by 1.753 times higher than that of a carbon cathode.^4^ Romo *et al.*^25^ explored the use of a biocathode in MFCs for Cr metal reduction using 454 pyrosequencing of the 16S rRNA gene; it was emphasised that removal of both the Cr metal and organic substrate was achieved using a biocathode with approximately 97% and 76% removal efficiency respectively. Cr metal reduction has been carried out in both double and single chamber MFCs, with higher efficiency and performance being achieved using double chamber MFCs compared with single chamber MFCs. For example, in a review of 18 models, a maximum performance of 1221.4 mW m^−2^ at 120 mg l^−1^, and 422.7 mW m^−3^ at 113.15 mg l^−1^ was observed for the double and single chamber respectively.^5,26^ While the application of MFC technology for heavy metal removal is known to offer numerous benefits, the challenge of scaling up these systems still poses a considerable obstacle to their widespread adoption.

Currently, sufficient information on the sustainability of MFC technology is lacking. Therefore, interest in evaluating the environmental and economic impacts of different bioelectrochemical systems has increased in recent years.^18,21,27^ A recent article by Sadhukhan *et al.*^20^ indicated that the life cycle assessment (LCA) of MFC technologies has not been sufficiently investigated, while there is no study directly focused on the environmental impacts of the use of MFCs for metal removal. Therefore, this study aims to fill this knowledge gap by evaluating the sustainability of metal removal from wastewater *via* the MFC technology, using hexavalent chromium (Cr(vi)) removal as a case study. This new study is expected to serve as an initial guide on the practicality of employing BESs for metal removal from an environmental point of view. It should also feed into future technological development and policy related to this technology. A detailed description of the MFC technology in relation to chromium metal removal is provided; the life cycle methodology and the effects of key variables were investigated in a sensitivity test.

## 2. Methodology

### a. Process description

**Fig. 1:**
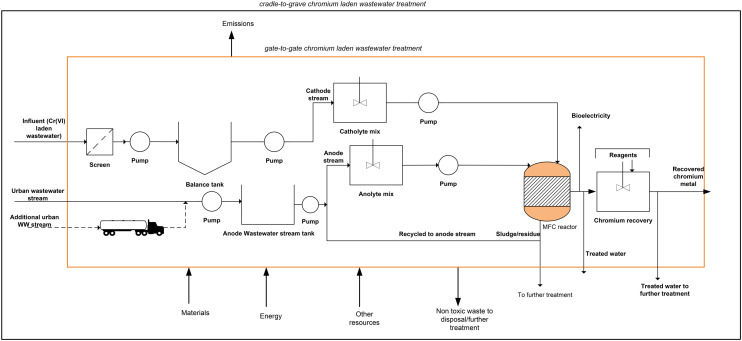
Process flow of chromium metal removal from wastewater *via* dual chamber MFCs.

A complete process for the chromium waste removal is shown in Fig. 1. Since most existing MFCs are currently at the laboratory^4,24^ or pilot scale^28,29^ conventional wastewater treatment that incorporates a dual chamber MFC reactor and subsequent auxiliaries was employed. The treatment plant was assumed to be located near a tannery site and the process starts with the receipt of chromium laden wastewater for the catholyte stream and urban wastewater for the anolyte stream.

The received tannery wastewater flows through pre-treatment units including screening to remove large and medium solid particles; the wastewater is then pumped to the balance tank to achieve a balance flow. The wastewater then undergoes pH correction in a pre-treatment tank for a residence time of 1 h. The chromium laden wastewater stream serves as the catholyte feed where hexavalent chromium (Cr(vi)) serves as the terminal electron acceptor. Since acidic pH is best for chromium reduction in the cathode chamber,^5,24^ hydrochloric acid (HCl) was used to implement an acidic pH for the catholyte. Urban wastewater serves as the anolyte feed, and a neutral pH is required in the anode chamber, hence, corrected *via* chemical dosing of citric acid and/or caustic soda (Fig. 1).

The wastewater undergoes treatment in a dual chamber MFC to reduce the toxic Cr(vi) to non-toxic Cr(iii) in the cathode chamber and simultaneously produce electricity. The Cr(iii) in solution is further precipitated in a separate tank, which is carried out by adjusting the pH to the optimum value of precipitation in the form of hydroxide by adding sodium or iron hydroxide (NaOH/FeOH) to obtain solid chromium at 99% efficiency. The amounts of reagents used were calculated stoichiometrically. The recovered chromium could displace the chromium obtained from virgin ore *via* conventional processes. Meanwhile, the generated electricity is assumed to be transformed to grid voltage which displaces the use of conventional grid electricity. The displaced burdens of materials and electricity were included in the LCA by system boundary expansion.

### b. MFC process

The MFC process modelled in this work was based on the laboratory experimental work by Li *et al.*^5^ on chromium removal and a pilot plant MFC process presented by Foley *et al.*^28^ A mass balance was carried out for the system using a basis of 1000 m^3^ daily influent of wastewater. Each unit operation from pre-treatment to post MFC units was modelled and sized using specific flows and operations.

The dual chamber MFC reactor consists of the anode and cathode chambers with an electrode immersed in the anolyte and catholyte medium respectively. The two chambers were separated by ion-exchange membrane. Heavy metal reduction in the MFC proceeds with three consecutive reactions in the order: 1) organic matter oxidation at the anode (electron production), 2) electron transfer in the external circuit, and 3) metal reduction at the cathode.^4^

**Fig. 2:**
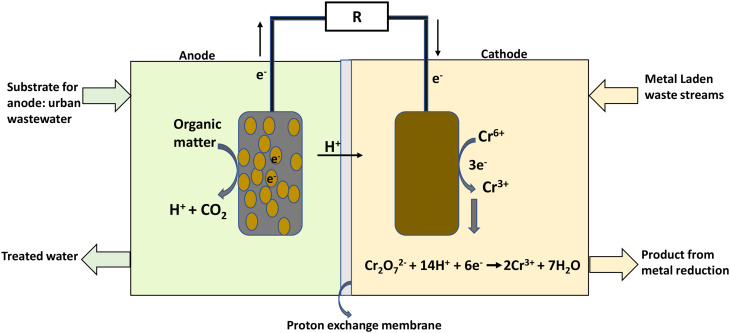
An illustration of the working principle of an MFC for hexavalent heavy metal removal (hexavalent Cr^6+^) [adapted from Li *et al.*^5^ & Sadhukhan *et al.*^20^].

Organic substrate oxidation by bacteria takes place in the anode chamber, producing electrons and protons in the process (eqn (1)). The electrons flow through an external circuit, while the protons move through the proton exchange membrane to the cathode chamber (Fig. 2).

1 C_6_H_12_O_6_ + 6H_2_O + microbes → 6CO_2_ + 24H^+^ + 24e^−^

Chromium reduction in the cathode chamber proceeds by harnessing the electrons and protons. Hexavalent chromium (Cr(vi)) has an oxidation reduction potential of 1.33 V (*vs.* standard hydrogen electrode; SHE) which requires six electrons for its reduction to Cr(iii); the Nernst equation shows the half-cell reaction potential (eqn (3)).

2 Cr_2_O_7_^2−^ + 14H^+^ + 6e^−^ → 2Cr^3+^ + 7H_2_O

3
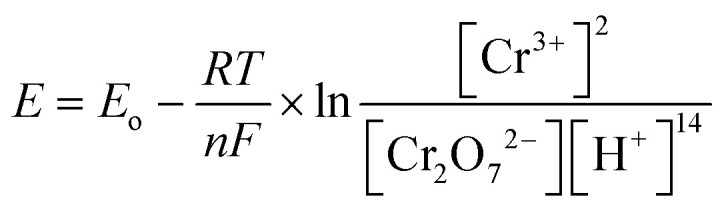
where *E*_o_ is the redox potential at the standard half-cell; *R* is the molar gas constant (8.31448 J mol^−1^ K^−1^); *T* is the temperature (K); *n* is the number of electrons exchanged, *i.e.*, 6, and *F* is Faraday's constant (96 485.3 C mol^−1^). The Cr(vi) reduction is thermodynamically favourable, allowing the electrons to flow spontaneously without external power,^4^ indicating that Cr(vi) can be considered as a terminal electron acceptor in the catholyte while harvesting electricity from the dual chamber MFC. Fig. 2 illustrates the working principle of a typical MFC used for hexavalent chromium reduction.

The bioelectricity generation and treatment of wastewater is dependent on the generated voltage, which is also influenced by Cr(vi) concentration, and pH of the catholyte,^5^ initial COD concentration, removal rate and applied potential. Eqn (4) was utilised in calculating the current density (A m^−3^) which was further used to determine the system's power (kW h) output.^30^ A conversion efficiency of 100% for Cr(vi) to Cr(iii), and a 95% recovery rate for chromium metal were assumed. A coulombic efficiency of 90% was employed for the base case. The dynamic behaviour involved in the Cr(vi) reduction process was incorporated in the LCA as discussed in further sections.4
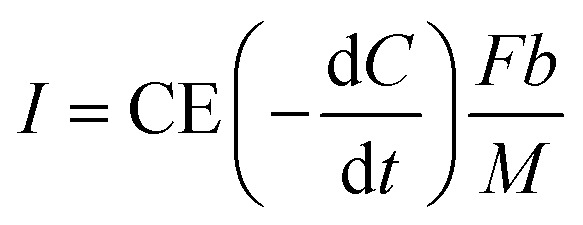
where 
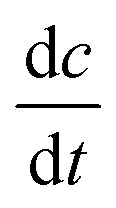
 [g m^−3^ h^−1^] is the COD removal rate, *F* is Faraday's constant, (96 485.3 C mol^−1^); *b* is the number of electrons produced per molecule of oxygen, 4; and *M* is the molar mass of oxygen, 32 g mol^−1^.

### c. Life cycle assessment

The LCA was carried out following the standard methodologies described in ISO 14040–14044.^31^ Four key interactive stages including goal and scope definition, life cycle inventory, life cycle impact assessment and interpretation were considered as discussed in the following sections.^31–33^ The SimaPro 9.4.0.2 software was used to conduct the assessment.^32^

#### i. Goal and scoping

The goal of the LCA study was to evaluate the life cycle environmental impact of removing hexavalent chromium from wastewater. A functional unit of 1 kg chromium recovered from the chromium wastewater treatment was defined. The system boundary utilised was ‘gate-to-gate’, which began with the receipt of chromium-laden wastewater, followed by chromium removal operations, and ended at the treatment plant gate prior to the reuse of the recovered chromium metal. A daily wastewater influent of 1000 m^3^ in a standard operating plant was assumed (section a), anode stream urban wastewater had a strength of 4400 mg COD L^−1^, while the cathode chromium laden wastewater stream had 95 mg L^−1^ chromium concentration.^34^ 50 m^3^ wastewater is recycled back to the anode stream from the anode outlet stream (Fig. 1); this adds up 1050 m^3^ per day for the anode inlet. These parameters served as the basis for inventory calculations.

#### ii. Life cycle inventory (LCI)

**Fig. 3:**
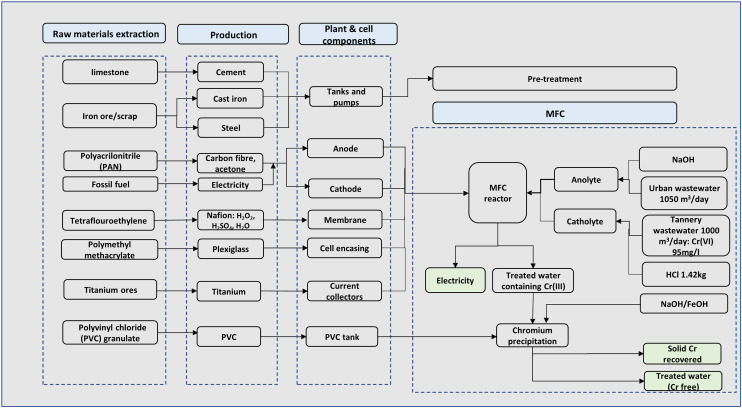
Materials and energy flows across the chromium waste treatment system boundary.

LCI is usually developed from process mass and energy balances, and environmental baseline data. Foreground data were based on existing standard information on wastewater treatment technologies by Tchobanoglous *et al.*^35^ and MFC work on chromium removal by Li *et al.*^5^ Background information consisting of all secondary data was obtained from the Ecoinvent database *via* the SimaPro 9.4.0.2 platform.^36^ The materials and processes associated with plant infrastructure, MFC components and operations are presented in Fig. 3. An MFC reactor size of approximately 400 m^3^ based on daily wastewater influent was used, and the same type of material (carbon cloth) was used for both cathode and anode electrodes. For each cell, a volume of 0.12 m^3^ per tube was utilised in calculating the mass of materials for each electrode based on previous studies.^28^ The influence of using alternative electrode materials was also investigated in a sensitivity test. A design life of 25 years was applied for the main plant infrastructure related to pre-treatment stages, while a variable design life was applied for MFC units due to data limitation on the extended design life for large scale MFC components. Cell encasing, membrane and reactor encasing were allocated a lifetime of 12 months, poly tanks including polyvinyl chloride (PVC) tanks used in the recovery unit were allocated a 10 year design life. For the membrane specifically, a one-year lifetime may seem brief under traditional circumstances. However, by assuming a shorter membrane lifespan, we aimed to consider the worst-possible scenario and assess the environmental impact of the process accordingly. We believe that this approach provides a more comprehensive understanding of the potential challenges and costs associated with the proposed treatment method under highly contaminated wastewater conditions. Operational aspects of the chromium removal are mainly reliant on chemical use and system efficiency, hence, energy use associated with the MFC was mainly in secondary processes and for material pre-treatments such as electrodes and membrane. Waste from the MFC is assumed to be minimal, 5% of solid waste (sludge) from the anode chamber is recycled back to the MFC while the remaining (including 100% from the cathode chamber) goes to solid treatment and disposal. Detailed inventory information is provided in the ESI.†

#### iii. Life cycle impact assessment

Life cycle impact assessment (LCIA) was used to evaluate the impact of removing and recovering chromium metal from wastewater *via* BESs. LCIA converts the long list of items in the inventory including all input and output data such as emissions, wastes, products and by-products, into a summarised range of impact indicators.^31^ The LCIA methodology adopted for this study was IMPACT 2002+ (v.2.14) *via* the SimaPro 9.4.0.2 platform.^36^ The IMPACT 2002+ has been used to evaluate the impact of some MFC processes for electricity generation^28^ and high value chemical production.^21^ This method covers both midpoint and endpoint impact indicators which are recognised as problem and damage oriented approaches respectively,^36^ as they look at the different stages in the cause–effect chain to calculate the impact. Although the endpoint impact category translates environmental impacts into issues of concern such as human health, and natural resources, its results have a higher level of uncertainty compared to midpoint.^37^ Midpoint categories are closely linked to inventory data for the LCA, and sources of environmental impact, making it easier to verify with lower uncertainty. These categories are also more commonly employed across various LCA studies which makes it easier for comparison with other studies.^38^ Thus, midpoint categories were utilised for the key evaluation and analysis. The main categories used in this study include global warming potential (GWP in kg CO_2_-eq.), non-renewable primary energy utilisation (NREU in MJ), carcinogens (kg C_2_H_3_Cl (chloroethylene)-eq.), ozone depletion (kg CFC-11 (chlorofluorocarbon)-eq.), terrestrial ecotoxicity (triethylene glycol equivalents into soil (TEG soil)-eq.), terrestrial acidification (kg SO_2_-eq.) and surplus mineral extraction (MJ). The 2016 ReCiPe Midpoint (M) Hierarchist (H) (1.02) impact assessment is a well-regarded globally relevant method.^36^ This impact assessment method has also been utilised in this work for comparison with the LCA study of chromium waste removal *via* conventional approaches. The specific impact categories selected from this method include global warming (kg CO_2_-eq.), terrestrial ecotoxicity (kg 1,4-dichlorobenzene (DCB)-eq.), human carcinogenic toxicity (kg 1,4-DCB-eq.), mineral resource scarcity (kg Cu-eq.), and fossil resource scarcity (kg oil-eq.).

#### iv. Sensitivity analysis

A sensitivity test was carried out on some of the key variables that influence the performance of the MFC process with regards to heavy metal removal to provide insights into the variability and significance of these parameters on the sustainability of the system. As shown in previous work, Cr(vi) concentration, initial COD concentration, removal rate, electron production and transfer efficiency greatly influence MFC output variables such as chromium recovered and bioelectricity generation and water quality. For example, Gadkari *et al.*^39^ highlighted the influence of substrate concentration on the voltage losses and expressed the concentration overpotential at the anode (*n*_conc) as a function of the initial substrate concentration (*S*_in) and the dynamic substrate concentration (*S*) as shown in eqn 5.5
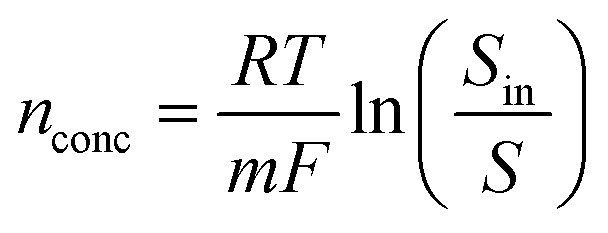
Since for every change in the initial chromium concentration in influent wastewater, the reduction reaction at the cathode could be limited by the initial COD concentration and/or the listed input variables. This implies that the total electrons required to reduce an nth mole of hexavalent chromium are affected by the change in electron production from substrate oxidation as a result of initial concentration. Therefore, the total converted and residual hexavalent chromium in the outlet stream for every 1 kg of chromium removed was estimated for all the tests. The base equations (eqn (1)–(3)) were used to conduct a quick check for the effect of varying initial COD concentration on chromium conversion, and consequently the LCA output. The effect of electrode type was investigated by Gadkari *et al.* (2021) in MES used for acetic acid production.^39^ The electrode sensitivity of the LCA output was also tested by replacing the carbon cloth electrode (Fig. 3) with a carbon brush. The materials used for carbon brush production as entered in SimaPro 9.4.0.2 are shown in the ESI.†^40^

For the sensitivity analysis, the factorial design is an established method for testing the sensitivity of the LCA output to various input variables.^37^ A fractional factorial design (2^4-1^) consisting of 8 runs and 4 variables was used as shown in Table 1. The 4 variables include the initial metal (Cr(vi)) concentration denoted as *C*, the initial substrate as COD concentration denoted with *S*, the electrode type denoted with *E*, and the coulombic efficiency denoted with *F* (Table 1).^41^ Two points (high and low) were selected for each variable based on the initial modelling results and previous studies on chromium removal *via* BESs.^5,15,34^ Statistical effects of the variables (C, S, E, F) and their interactions (*e.g.*, CS, CE, SF, CSE, SEF) on the responses (life cycle environmental impacts) were calculated based on the model results. Effects are estimated as the differences between the averages for the high and low levels of a variable or interaction, and the total mean response.^41,42^ The highest order interactions of variables (*e.g.*, CSEF) were assumed to be largely due to random error, hence not shown in the analysis. Normal probability plots of the effects were used to visualize the significance of the effects of individual variables and their interactions on the impacts (Fig. 9). The estimated effects can be read from the *X*-axis against the standard deviation of the normal distribution on the *Y*-axis.^42^ The *Y*-scale has been altered such that a normal distribution is shown as a straight line, and points that fit onto this line may be a result of normal random variability, while those that deviate from the straight line show significant effects of the variables and their interactions.

**Table 1:** Sensitivity analysis results of LCA chromium waste removal

Variables					Output (per kg chromium recovered)			
S/NO	Cr(vi) concentration (mg l^−1^) (*C*)	Initial COD (mg l^−1^) (*S*)	Electrode (type) (*E*)	Coulombic efficiency (%) (*F*)	GWP (kg CO_2_-eq.)	NREU (MJ)	Terrestrial Ecotoxicity (kg TEG soil)	Mineral extraction (MJ surplus)
1	95	800	Carbon cloth	50	2.81	44.3	130	−1.01
2	150	800	Carbon cloth	80	−2.89	−47.6	3.53	−1.65
3	95	4400	Carbon cloth	80	0.026	8.82	95.2	−1
4	150	4400	Carbon cloth	50	−3.98	−61.8	−10.5	−1.65
5	95	800	Carbon brush	80	1.65	15.4	163	−0.917
6	150	800	Carbon brush	50	−3.44	−66.1	51	−1.49
7	95	4400	Carbon brush	50	−0.088	−6.32	142	−0.917
8	150	4400	Carbon brush	80	−5.96	−101	12.2	−1.56

Eqn 6 represents the fitted model for the predicted responses and eqn (7) was used to calculate the residuals (*ε*) of the responses.^41^ Analysis of variance (ANOVA) was also used to evaluate the statistical significance of the estimated effects. It is also worth noting that positive main effects increase a response when the settings change from the low value of a factor to the high value and negative main effects decrease the response when the settings change from the low value of a factor to the high value. In terms of environmental assessment, the lower the effect the better the results as it relates to environmental savings.6
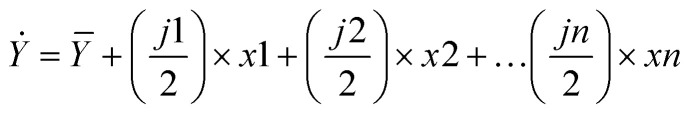

7 *ε* = *y* − *Ẏ*

where *Ȳ* is the grand mean for each set of response data (*e.g.*, GWP (kg CO_2_-eq.)); *j*1, *j*2… *jn* is the observed main or interaction effect of the variables; *x*1, *x*2… *xn* is the respective sign of the observed effects for each response value.

## 3. Results and discussion

### a. Life cycle impact of chromium metal recovery

**Fig. 4:**
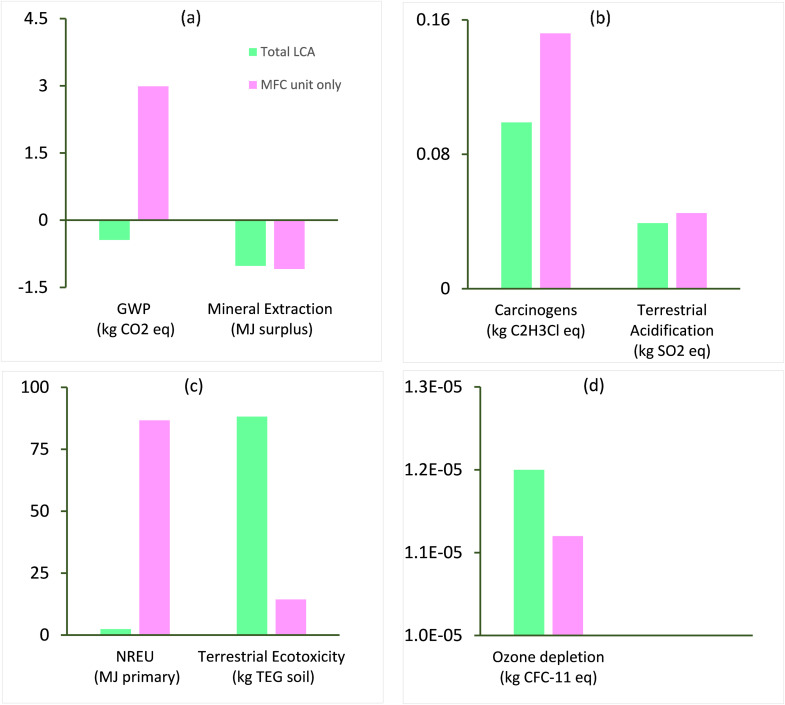
Environmental impact of chromium waste removal: a) GWP (kg CO_2_-eq.) & mineral extraction (MJ surplus), b) carcinogens (kg C_2_H_3_Cl-eq.) & terrestrial acidification (kg SO_2_-eq.), c) NREU (MJ primary) & terrestrial ecotoxicity (kg TEG soil) d) ozone depletion (kg CFC-11-eq.).

The environmental life cycle impact characterisation of a substance in an impact category is the change in its intrinsic properties responsible for the category due to the change in its abundance in the environment with respect to the change of a reference substance. This section evaluates the environmental impact of removing 1 kg of chromium metal from a wastewater stream for the specific system boundary described in section 2.c.i. Fig. 4(a–d) show the characterisation results for the total LCA of chromium waste removal compared with the LCA of a specific MFC unit for impact categories including global warming potential (GWP, kg CO_2_-eq.), mineral extraction (MJ surplus), carcinogens (kg C_2_H_3_Cl), terrestrial acidification (kg SO_2_-eq.), non-renewable energy use NREU (MJ primary), terrestrial ecotoxicity (kg TEG soil), and ozone layer depletion (kg CFC-11-eq.). The terrestrial ecotoxicity (kg TEG soil) and NREU (MJ primary) impact of the total LCA and specific MFC unit aligns with a similar level of impact reported in previous works on BESs^21,43^ and may be attributed to the specific type of energy used in the process and energy related to material sourcing for MFC components. Environmental savings in terms of GWP (kg CO_2_-eq.) was observed for the total LCA, while a high environmental burden GWP (kg CO_2_-eq.) was associated with the specific MFC unit. The end of life (EoL) scenario (recycling) of the various materials of construction such as polyvinyl chloride and polymethyl methacrylate (PMMA) reduced the net global warming potential of the system. However, the specific MFC unit without EoL and other units showed high impact as a result of CO_2_, CH_4_ and N_2_O emissions from fossil fuels (*e.g.*, diesel, coal) in the initial stages of the life cycle (Fig. 4). The remaining impact categories including ozone depletion, carcinogens and terrestrial acidification had relatively minimal impact on the total life cycle of the chromium waste removal.

Due to the lack of information on the LCA of conventional treatments for recovering chromium from wastewater, it is challenging to compare the findings of the current study with existing works. Additionally, there is no available research on the LCA of chromium recovery *via* BESs, making it even harder to draw comparisons. Existing studies on the LCA of chromium waste removal incorporated the chromium laden wastewater treatment as part of a chrome plating system.^44^ The few studies on the LCA of chromium laden wastewater treatment reported higher impact in terms of GWP compared with values obtained in this study (−0.44 kg CO_2_-eq. per kg Cr recovered). For example, Rodríguez *et al.*^13^ reported 0.15 kg CO_2_ eq. per m^2^ of chromium used to coat a piece of material using alternative technologies with key units including ion exchange and photocatalysis, while the same authors compared their results with a value of 1.5 kg CO_2_-eq. per m^2^ of treated piece for conventional treatment units including reduction, precipitation and settling. Vidal *et al.*^44^ reported a GWP of 0.38 kg CO_2_-eq. per m^2^ of surface treated for treatment of chromium laden wastewater generated from plastic (acrylonitrile butadiene styrene) metal plating. These studies employed the ReCiPe (M) (H)^36^ methodology for impact assessment, hence, for comparative purposes, the ReCiPe (M) (H) methodology was used in the current study to obtain a value of 0.8 kg CO_2_-eq. for 1 kg chromium recovered which is still lower than normalised values of 2.88 and 28.8 kg CO_2_-eq. obtained by Rodríguez *et al.*^13^ for 1 kg of chromium removal. Our results indicate a total savings on GWP of 72% and 97% respectively.

The low GWP impact achieved in the current work can be attributed to the avoidance of electricity usage, environmental credits earned from end-of-life treatments of the MFC unit's construction materials, and the recovery of chromium. However, a GWP of 11 kg CO_2_-eq. was obtained before allocation of avoided burdens of the chromium waste recovery process. This value appears to be much higher than the alternative treatment proposed by Rodríguez *et al.*^13^ but still lower than the conventional treatment which had 28.8 kg CO_2_-eq. presented by the same authors. The high impact of the MFC on GWP is associated with reactor materials such as polymethyl methacrylate (PMMA) and tetrafluoroethylene (TFE), which further emphasises the need to develop more sustainable materials of construction for MFC reactor components.

Furthermore, the life cycle environmental impact of the MFC system on terrestrial ecotoxicity (Fig. 4), which is dominated by emissions of chemicals such as pesticides to soil, may be linked to upstream processes of materials and energy production; this observation is in line with other studies for conventional wastewater treatment^45^ and MES.^28^

### b. Contribution of chromium waste treatment stages

**Fig. 5:**
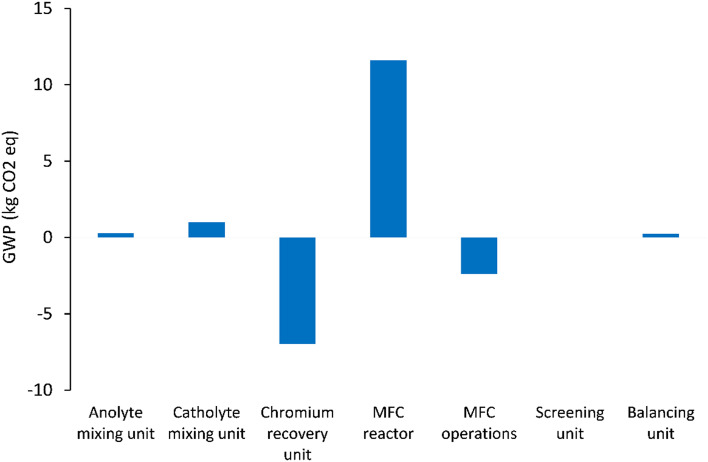
GWP (kg CO_2_-eq.) impact of units considered in the chromium waste treatment (MU = mixing unit; RU = recovery unit).

**Fig. 6:**
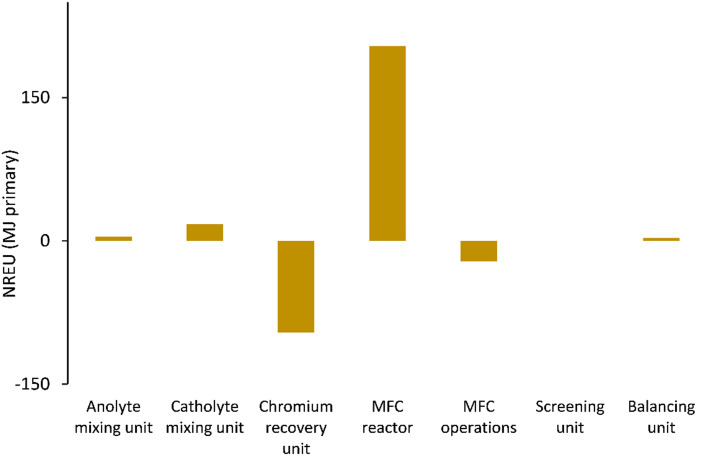
Mineral extraction (MJ surplus) impact of units considered in the chromium waste treatment (MU = mixing unit; RU = recovery unit).

**Fig. 7:**
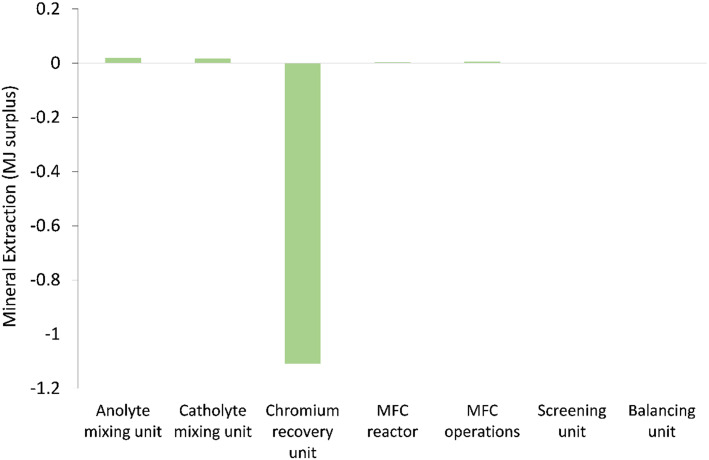
NREU (MJ primary) impact of units considered for chromium waste treatment (MU = mixing unit; RU = recovery unit).

**Fig. 8:**
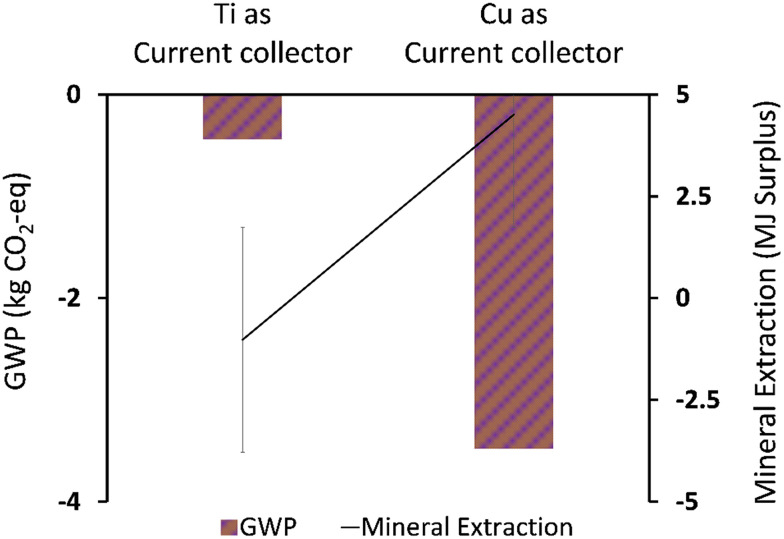
Life cycle environmental impact of titanium (Ti) *vs.* copper (Cu) used as a current collector.

Fig. 5–7 show the life cycle environmental impact of various components of the chromium (Cr(vi)) waste treatment process. For all the units considered, the chromium recovery unit had the least impact across all the categories including GWP (kg CO_2_-eq.), NREU (MJ primary), and mineral extraction (MJ surplus), which may be attributed to the avoided burden of chromium production allocated to this unit. The MFC operation also indicates significant environmental benefits with a negative value of −2.4 kg CO_2_-eq. for GWP and −21.6 MJ primary for NREU and an environmental impact of 0.0057 MJ surplus for mineral extraction. The allocated credit of bio-electricity generation by the MFC unit and very minimal operational energy requirement contributes to the observed environmental benefits. As demonstrated in other related studies,^28,39^ the net energy potential of an MFC system (depending on the terminal electron acceptor) makes it an attractive and promising technology for sustainable bioenergy and waste management. Hexavalent chromium used as the terminal electron acceptor has a good positive redox potential of 1.33 V (*vs.* standard hydrogen electrode, SHE).^1,5^ The base case had electricity generation of 4.46 kW h for each 1 kg of chromium recovered, with an estimated current density of 216.4 A m^−3^ at 90% coulombic efficiency. The system was assumed to have minimal waste generation,^4,5^ hence, the MFC unit had a relatively small operational energy burden. On the other hand, the MFC reactor appeared to be the most intensive unit across all categories. The impact on GWP and NREU (Fig. 5 and 7) was mostly produced by raw material procurement and electricity consumption during manufacturing. Materials such as polymethyl methacrylate (PMMA) used for MFC cell encasing and reactor external walls, as well as tetrafluoroethylene (TFE) used for the cell membrane, indicate extremely high impact on GWP (Fig. 5). Previous studies have also highlighted the intensity of materials of construction for MFC reactors.^21^ Further research is therefore imperative to develop alternative and more sustainable materials for MFC reactor components and other BES technologies. The effect of electrode materials such as carbon cloth sourced from polyacrylonitrile (PAN) was slightly overshadowed by the impact of the main reactor encasing. However, current collectors such as titanium connecting the two electrodes showed a significant effect on the system's environmental impact. Replacing titanium with copper as the current collector significantly further reduced the GWP of the overall system as can be seen in Fig. 8.

This implies the unsuitability of titanium for use in commercial scale MFCs. Interestingly, it can be observed that changing the current collector had a big swing on mineral extraction (MJ surplus) (Fig. 8), replacing titanium with copper increased the mineral extraction (MJ surplus) impact. This may be attributed to a high negative value contributed by some of the processes along the titanium production chain. Bauxite mine operations and chromite ore concentrate production indicate significant reduction in the resource extraction (MJ surplus) which was a secondary process in the model with titanium. Meanwhile, copper sulfide mining indicates an increase in mineral extraction impact but significant reduction in GWP, since the two categories focused on different elements along the cause–effect chain. Error bars were used to check for statistical significance of the big swing in the mineral extraction impact of the two current collectors. As can be seen, a small overlap indicates a real difference between the two data sets for the mineral extraction category. Further evaluation of some of the key model parameters is performed in a sensitivity test.

### c. Sensitivity analysis

The 2^4-1^ fractional factorial matrix used in the sensitivity tests and the corresponding responses as life cycle environmental impacts is shown in Table 1. For all the eight runs carried out, the interaction of carbon brush, lower concentration of COD and lower concentration of Cr(vi) yielded the highest environmental impact across the three impact categories considered in the sensitivity test. Meanwhile, an interaction of carbon brush with higher levels of the remaining three variables had the least environmental impact in terms of GWP and NREU. It can also be observed that the Cr(vi) concentration has a strong influence on the overall results as it overshadows the effect of changing other variables (*e.g.*, COD) on the LCA output.

The two points selected for COD in Table 1 yielded a sufficient number of electrons (from substrate oxidation) to complete chromium reduction at 100% conversion efficiency. The higher initial COD concentration increased the electricity generation and subsequently reduced the environmental impact while the higher level of initial metal concentration (Cr(vi)) increased the environmental burden; however, the credit allocation for chromium metal and electricity generation significantly outweighs the environmental burden. Furthermore, from contribution analysis, the credit allocation for chromium recovered showed stronger impact on the LCA results compared with credit allocation for electricity for the specific case study. This observation may change when other factors such as substrate type, bacteria species, their performance, and interaction with the electrodes are incorporated in the sensitivity tests. Meanwhile, electricity generation was consistent with the change in COD concentration and coulombic efficiency; likewise, the total recovered Cr metal changed for all the test combinations, and any residual Cr(vi) was considered an emission to water within the input inventory.

Out of all the variables considered in Table 1, the initial metal concentration (Cr(vi)) seems to have the most sensitive effect under the given conditions. Existing works on chromium metal removal have highlighted the influence of the initial metal concentration on hexavalent chromium reduction in MFCs.^4,15^ For example, Li *et al.*^5^ in their work observed an improvement in the maximum power density and open circuit potential to be closely related to the initial Cr(vi), which was linked to the high redox potential of Cr(vi) as utilised in the Nernst equation. The same authors also indicated that the reduction reaction on cathodes could be accelerated at higher Cr(vi) concentration, which was in accordance with the lower internal resistance at higher Cr(vi) concentration.

**Fig. 9:**
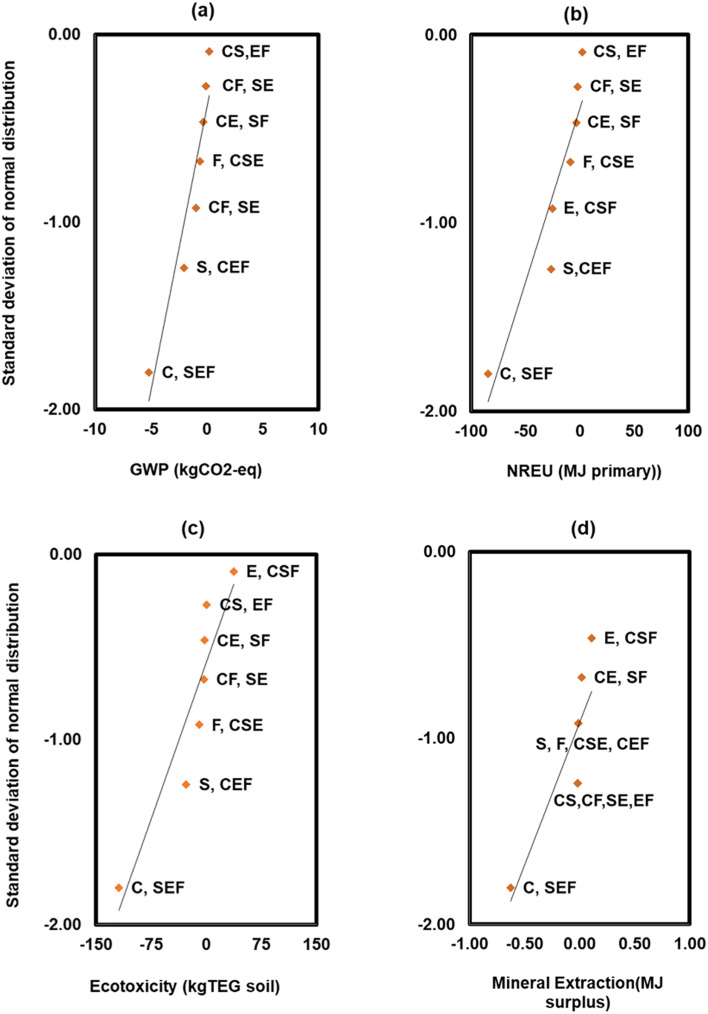
Normal probability plots of the main and interaction effects of chromium concentration (C), substrate (COD) concentration (*S*), electrode (E), and coulombic efficiency (*F*) on life cycle environmental impacts (see 2.c.iv for definition of interaction codes).

The normal probability plot of the main and interaction effects of the observations from Table 1 is shown in Fig. 9a–d. The effects that deviate from the straight lines in the probability plots are the most significant.^46^ The magnitudes of the effects, and the probabilities that they are attributable to random error, *p*-value, were determined based on the *F*-statistics calculated in the analysis of variance (ANOVA). An effect is considered as statistically significant when *p* < 0.05.^41^

From Fig. 9, an increase in the initial metal concentration (hexavalent chromium) (C) exhibited a significant negative main effect (*p* = 0.0001) on all the impact categories evaluated in the sensitivity test, which can be translated to environmental savings as the lower the value the better the environmental profile and aligns with the observation in Table 1. However, a two-factor interaction of chromium and COD (CS), and a separate two factor interaction of electrode and coulombic efficiency (EF), did not yield a significant interaction effect on GWP (*p* = 0.43; *p* = 0.44) and NREU (*p* = 0.53; *p* = 0.56) respectively. Furthermore, increasing the COD (S) showed a negative main effect on GWP (*p* = 0.003), NREU (*p* = 0.002), and terrestrial ecotoxicity (*p* = 0.002) which indicates reduction in environmental impacts, and translates to environmental savings. The higher level of initial substrate concentration increased the total electron production, which influences the electricity production of the system,^4,39^ and consequently credits allocation over the life cycle of the recovered chromium. This effect was slightly confounded with that of the initial metal concentration because of the combinations used in Table 1. A three-factor interaction of COD, electrode, and coulombic efficiency (SEF) did not yield any clear effect on any of the impact categories with the least observed magnitude (*p* = 0.74). Likewise, the three-factor interaction of chromium, substrate, and electrode (CSE) did not yield any apparent effect on GWP (*p* = 0.33), NREU (*p* = 0.24) and mineral extraction (*p* = 0.33). However, previous work by other authors indicated the significant influence of these variables on chromium reduction^5^ and production of high value chemicals *via* BESs,^21,43^ which shows the importance of combined optimisation of these variables. It was also observed that replacing carbon cloth with a carbon brush electrode did not result in a substantial change in GWP and NREU which may be attributed to the influence of other variables such as metal chromium and substrate initial concentrations. The main effect of coulombic efficiency (*F*) was not significant on GWP (*p* = 0.11) and NREU (*p* = 0.86) but slightly reduced the ecotoxicity impact (*p* = 0.02). This is partly because the two levels of coulombic efficiency utilised in the sensitivity test was greatly overshadowed by the effects of other variables.

Interestingly, only the main effect of electrode and chromium concentration had a significant main negative and main positive effect on mineral extraction (*p* = 0.0001) and (*p* = 0.012) respectively. In this context, the negative values indicate reduction in the environmental impact while the positive values show an increase in the environmental impact. This further emphasised the positive correlation between higher initial chromium and improved power production of the MFC and recovered chromium metal, depending on the system's efficiency. The effects of electrode and chromium on surplus mineral extraction may be attributed to mineral production processes related to electrode production and recovered chromium metal. It can also be highlighted that in terms of operating variables, the concentration of the metal to be recovered plays a key role not only in the MFC's performance, but also in the environmental implications of recovering metals *via* BESs.

## 4. Conclusion

This study investigated the life cycle environmental impact of using microbial fuel cell systems for simultaneous hexavalent chromium waste metal recovery and electricity generation. The results demonstrate the suitability of employing bioelectrochemical systems such as MFCs for heavy metal removal and recovery. The MFC had a positive performance with an estimated bioelectricity production of 4.4 kW h and estimated current density of 216.4 A m^−3^ for 1 kg of chromium recovered. The LCA results show a favourable sustainability profile of the chromium waste recovery *via* a MFC with a GWP impact of −0.44 kg CO_2_-eq. (∼0.8 kg CO_2_-eq. (using the ReCiPe methodology)), which was substantially better in comparison with the LCA of conventional chromium removal technologies (2.8 kg CO_2_-eq. for ion exchange and photocatalysis, and 28.8 kg CO_2_-eq. for chemical reduction–precipitation–settling).

For all the different units considered within the chromium waste treatment plant, the MFC unit itself had the highest impact in terms of GWP (kg CO_2_-eq.) and NREU (MJ primary) while the chromium recovery unit had the highest percentage contribution to the impact on mineral extraction (MJ surplus). The operational burden of the MFC system was very minimal due to the positive energy balance of the process resulting from the favourable redox potential of hexavalent chromium, and adequate substrate concentration. However, the GWP (kg CO_2_-eq.) and NREU (MJ primary) impacts were dominated by the construction burden of the system, in particular material acquisition for MFC reactor components was significantly high with 11 kg CO_2_-eq. while the chromium recovery unit had the least impact of −6.9 kg CO_2_-eq. for GWP, and −1.11 MJ surplus for mineral extraction. It was also observed that the use of titanium for cell components is currently not environmentally viable for large scale application of MFCs for metal recovery. The output of the LCA was highly sensitive to small changes in the materials of construction and associated processes. This implies a need for further research in this area to develop alternative sustainable materials. It would also be interesting to compare the LCA output of various reactor materials of construction.

Among the four system variables studied in the sensitivity analysis including initial chromium concentration, substrate COD, electrode, and coulombic efficiency, the chromium concentration had the most noticeable impact. A statistical analysis of the results showed that the higher concentration of chromium was associated with significant reduction in environmental impact for all impact categories. However, there was no apparent interaction effect of chromium with the rest of the variables on the LCA output. A high substrate (COD) also indicates environmental savings on GWP (kg CO_2_-eq.), NREU (MJ primary) and terrestrial ecotoxicity (MJ surplus). Further work should focus on understanding the effects of other variables, such as dynamic microbial activity, changes in removal efficiency, and alternative materials for MFC reactor components, as well as other types of metals, on the environmental and economic performance of metal removal *via* MFCs.

## Author contributions

Rukayya Ibrahim Muazu: conceptualization, methodology, software, resources, validation, formal analysis, investigation, data curation, writing – original draft preparation. Jhuma Sadhukhan: conceptualization, funding acquisition, writing, review and editing, supervision, project administration. S. Venkata Mohan: funding acquisition, writing, review and editing. Siddharth Gadkari: conceptualization, funding acquisition, software, writing, review and editing, visualization, supervision, project administration.

## Conflicts of interest

The authors declared that they have no financial and personal relationships with other people or organizations and potential conflicts of interest in this work. The funders had no role in the design of the study; in the collection, analyses, or interpretation of data; in the writing of the manuscript, or in the decision to publish the results.

## Supplementary Material

EW-009-D3EW00344B-s001
