# Supplementary material for: Hexavalent chromium waste removal via bioelectrochemical systems – a life cycle assessment perspective
Source: Environ Sci (Camb). 2023 Jul 31;9(10):2487–500. doi: 10.1039/d3ew00344b (PMC10534287; doi:10.1039/d3ew00344b)
Supplement: EW-009-D3EW00344B-s001 [file EW-009-D3EW00344B-s001.pdf]

Electronic Supplementary Material (ESI) for Environmental Science: Water Research & Technology.
This journal is © The Royal Society of Chemistry 2023

Supplementary Information:

Table A1: Life cycle inventory of hexavalent chromium waste removal

FU=functional unit (1 kg chromium metal recovered)

**Chromium Waste Treatment Stage**			**Process used**	**Notes**
Pre-treatment				
**Screening**	**kg/FU**	**kW/FU**		
Concrete	0.0125432		Concrete, normal {CH}| unreinforced concrete production, with cement CEM II/B | APOS, U; Concrete, high exacting requirements {CH}| concrete production, for building construction, with cement CEM II/B | APOS, U	3% reinforcement was applied and allocated to high exacting requirements. The process "Inert waste, for final disposal {CH}| market for inert waste, for final disposal | APOS, U" was used for all concrete/construction waste treatment.
Steel	0.0002726		Steel, chromium steel 18/8 {RER}| steel production, converter, chromium steel 18/8 | APOS, U	
Energy const.		0.0001103	Electricity mix, AC, consumption mix, at consumer, 1kV - 60kV GB S	
Balancing	**kg/FU**	**kW/FU**		
Concrete	0.2686278		Concrete, normal {CH}| unreinforced concrete production, with cement CEM II/B | APOS, U; Concrete, high exacting requirements {CH}| concrete production, for building construction, with cement CEM II/B | APOS, U	
Energy const.		0.002192	Electricity mix, AC, consumption mix, at consumer, 1kV - 60kV GB S	
Pump material (Cast iron)	0.0011376		Cast iron {RER}| production | APOS, U	

Operations pump		0.343257	Electricity, high voltage {GB}| production mix | APOS, U	
Anolyte feed prep	**kg/FU**	**kW/FU**		
Concrete	0.0189399		Concrete, normal {CH}| unreinforced concrete production, with cement CEM II/B | APOS, U; Concrete, high exacting requirements {CH}| concrete production, for building construction, with cement CEM II/B | APOS, U	
Steel mixer	0.0039155		Steel, chromium steel 18/8 {RER}| steel production, converter, chromium steel 18/8 | APOS, U	
Tank Pumps material (Cast iron)	0.0017		Cast iron {RER}| production | APOS, U	
Energy const.		0.0003859	Electricity grid mix 1kV-60kV, AC, consumption mix, at consumer, 1kV - 60kV GB S	
Operations		0.4676139	Electricity, high voltage {GB}| production mix | APOS, U	
Catholyte feed prep	**kg/FU**	**kW/FU**		
Concrete	0.0021325		Concrete, normal {CH}| unreinforced concrete production, with cement CEM II/B | APOS, U; Concrete, high exacting requirements {CH}| concrete production, for building construction, with cement CEM II/B | APOS, U	
Steel mixer	0.0011101		Steel, chromium steel 18/8 {RER}| steel production, converter, chromium steel 18/8 | APOS, U	
Energy const.		0.000085	Electricity grid mix 1kV-60kV, AC, consumption mix, at consumer, 1kV - 60kV GB S	
Tank Pump material (Cast iron)	0.0014		Cast iron {RER}| production | APOS, U	

Operations		0.1170081	Electricity, high voltage {GB}| production mix | APOS, U	
Chemical(HCl)	1.42		Hydrochloric acid, without water, in 30% solution state {RER}| market for | APOS, U	
MFC reactor MoC	**kg/FU**	**kW/FU**		
Encasing	0.84		Polymethyl methacrylate, sheet {RER}| production | APOS, U	
Membrane	0.0026		Tetrafluoroethylene {RER}| production | APOS, U	
Anode (Carbon fibre)	0.162		Polyacrylonitrile fibres (PAN), from acrylonitrile and methacrylate, prod. mix, PAN w/o additives EU-27 S; Electricity, high voltage {GB}| electricity production, natural gas, combined cycle power plant | APOS, U; Transport, freight, lorry 7.5-16 metric ton, EURO5 {RER}| transport, freight, lorry 7.5-16 metric ton, EURO5 | APOS, U	losses in manufacturing was assumed to be the same for all the electrodes
Cathode (Carbon fibre)	0.162		Polyacrylonitrile fibres (PAN), from acrylonitrile and methacrylate, prod. mix, PAN w/o additives EU-27 S; Electricity, high voltage {GB}| electricity production, natural gas, combined cycle power plant | APOS, U; Transport, freight, lorry 7.5-16 metric ton, EURO5 {RER}| transport, freight, lorry 7.5-16 metric ton, EURO5 | APOS, U	

Electrode carbon brush	0.162		Graphite {GLO}| market for | APOS, U; Petroleum coke {GLO}| market for | APOS, U; Zinc {GLO}| market for | APOS, U; Polyphenylene sulfide {GLO}| market for | APOS, U; Extrusion, plastic pipes {RER}| production | APOS, U	Process energy for plastic extrusion was utilised for brush.
Electrode surfacing	0.0042345		Polymethyl methacrylate, sheet {RER}| production | APOS, U	Input in simapro was combined with carbon fibre production from PAN
Cell component	0.0051962		Polymethyl methacrylate, sheet {RER}| production | APOS, U	
Current collector	0.12		Titanium, primary {GLO}| market for | APOS, U	
Current collector	0.12		Copper {RER}| production, primary | APOS, U	
Electrode heat treatment		0.0357845	Electricity, high voltage {GB}| production mix | APOS, U	
Chemicals:H2O2	0.3371495		Hydrogen peroxide, without water, in 50% solution state {RER}| hydrogen peroxide production, product in 50% solution state | APOS, U	
Chemicals:H2SO4 (0.5 M)	0.4255059		Sulfuric acid, at plant/kg/RNA	
Water: De-ionised water	0.291703		Water, deionised, from tap water, at user {Europe without Switzerland}| water production, deionised, from tap water, at user | APOS, U	
Chemicals: Acetone	0.1828838		Acetone, liquid {RER}| production | APOS, U	
Waste treatment	0.0290141		Sewage sludge {GLO}| market for | Conseq, U	
Avoided Electricity		4.4	Electricity, high voltage {GB}| production mix | APOS, U	

Chromium metal recovery unit	**kg/FU**			
Tank material (PVC)	0.7559196		Polyvinyl chloride resin, at plant/RNA; Polyethylene, high density, granulate {CH}| polyethylene, high density, granulate, recycled to generic market for high density PE granulate | APOS, U; Blow moulding {GLO}| market for | APOS, U; Steel, chromium steel 18/8, hot rolled {RER}| production | APOS, U	The assumed recycled content-high density polyethylene (HDPE) was used in place of PVC, while virgin PVC remains unchanged
Chemicals: FeOH or NaOH	1.1236257		Sodium hydroxide, without water, in 50% solution state {RER}| chlor-alkali electrolysis, diaphragm cell | APOS, U	
Chromium metal recovered	1		Chromium {RER}| production | APOS, U	Assumed for recovered metal
End of Life (EoL) for process materials	**kg/FU**			
Total MoC waste	7.31		Mixed plastics (waste treatment) {GLO}| recycling of mixed plastics | APOS, U; Waste polyethylene, for recycling, sorted {Europe without Switzerland}| treatment of waste polyethylene, for recycling, unsorted, sorting | APOS, U; Polyethylene, high density, granulate, recycled {Europe without Switzerland}| polyethylene production, high density, granulate, recycled | APOS, U	A net scrap approach was used for some of the materials specifically PVC as it has the highest percentage share of the waste and better data on recycling and product information. Waste PE data was also used in some instances as reference material for the plastic components.
